# Phenotypic Description of the Spanish Multicentre Genetic Glaucoma Group Cohort

**DOI:** 10.1155/2017/1907454

**Published:** 2017-09-26

**Authors:** Elena Milla, Maria José Gamundi, Susana Duch, Jose Rios, Miguel Carballo, EMEIGG Study Group

**Affiliations:** ^1^Unidad de Glaucoma, Institut Clínic d'Oftalmologia (ICOF), Hospital Clínic, Barcelona, Spain; ^2^Unidad de Glaucoma y Genética, Institut Comtal d'Oftalmologia, Barcelona, Spain; ^3^Servicio de Laboratorio, Hospital de Terrassa, Barcelona, Spain; ^4^Servicio de Estadística, Hospital Clinic, Barcelona, Spain; ^5^EMEIGG, Spanish Multicentre Glaucoma Group (Estudio Multicéntrico Español de Investigaciones Genéticas en Glaucoma), Spain

## Abstract

**Introduction:**

The aim of the study was to make a phenotypic description of the Spanish multicentre glaucoma group cohort of patients.

**Design:**

Retrospective, observational, multicentre, cohort study.

**Material and Methods:**

The clinical charts of 152 patients with hereditary glaucoma from18 Spanish eye centres were reviewed in order to make an epidemiologic description of the type of glaucoma and associated factors. True hereditary cases were compared with familiar cases according to the Gong et al. criteria.

**Results:**

61% were true hereditary cases and 39% familiar cases. Ocular comorbidity, optic disc damage, and visual field mean defect were significantly more severe in hereditary patients, who required significantly more first-line hypotensive drugs and surgical interventions to control intraocular pressure than familiar patients.

**Conclusions:**

The strength of the hereditary component of glaucoma seems to worsen the clinical course, causing more structural and functional damage and requiring more intense glaucoma treatment. The family history of glaucoma should be carefully investigated and taken into consideration when making treatment decisions or intensifying previous treatment.

## 1. Introduction

By 2020, 79.6 million people will have glaucoma or ocular hypertension (OHT) and, of these, 74% will have primary open angle glaucoma (POAG). Glaucoma will continue to be the second leading cause of blindness worldwide [1]. In POAG, early detection and treatment is effective in reducing disease progression [2]. However, 50% of cases are known to be undiagnosed. The two main reasons for underdetection of glaucoma are poor uptake of community eye care services and cases missed by primary eye care services [3]. Due to the public health importance of glaucoma, it has been suggested as a condition that might merit population-based screening programs.

However, as Lander and Shork stated, glaucoma is a complex trait and, in the majority of cases, does not follow a clear-cut inheritance pattern, has a variable penetrance transmission and an insidious progression, and usually has a late onset [4].

Paradoxically, other hereditable ophthalmic pathologies, such as retinitis pigmentosa (RP), are far less common but their genetic profile has been much better characterized [5]. The difference between RP and glaucoma is that the latter is treatable if detected early [6–9]. Therefore, the development of an accurate test to detect presymptomatic carriers at risk is important for the management of glaucomatous optic neuropathy (GON).

Despite considerable research efforts, the aetiology of POAG remains unknown, probably due to the heterogeneous and complex clinical and molecular nature of the disease. In the last decade, much effort has gone into elucidating the genetic causes and risk factors of POAG. The application of advanced genetic technology, such as Genome-Wide Association Studies (GWAS), large-scale gene expression studies, and proteomics, has yielded a wealth of information. A large amount of candidate POAG disease genes have been identified, and there is increasing insight into the molecular mechanisms underlying POAG [10]. Unfortunately, the underlying molecular mechanisms cannot be elucidated in the majority of glaucoma cases, although a familiar component is clearly present in a large number.

In 2009, our research group initiated a prospective multicentre study which collected blood samples from patients with familial and hereditary glaucoma throughout Spain in order to carry out genetic characterization with the aim of describing the mutational profile of glaucoma in Spain. After the results were published [11], we assessed the phenotypic data of the study population to characterize the clinical profile of this sample of Spanish glaucoma patients. This is the first description of the phenotypic mapping of glaucoma in Spain.

Therefore, the objective of this study was to describe the epidemiologic features of patients included in our database.

## 2. Patients and Methods

Before study inclusion, all participants were informed of the study objectives. All gave informed consent to participate in the study, which adhered to the tenets of the Declaration of Helsinki. All were asked to complete a questionnaire that included personal, biographic, demographic, family, and clinical data.

This study was approved by the Ethics Committee of the Hospital Clinic of Barcelona.

Patients from 18 Spanish hospitals were enrolled by their treating ophthalmologists in a collaborative study named Estudio Multicéntrico Español de Investigación Genética del Glaucoma (EMEIGG). We did not include the total EMEIGG population in the present study. An equitable proportion of patients from each eye centre were included in order to homogenize the results between the different Spanish regions.

Patients were enrolled consecutively by their glaucoma specialist if they accomplished the inclusion criteria described below.

Inclusion criteria were POAG patients diagnosed with familiar or hereditary glaucoma and sporadic single patients (without affected relatives) with congenital, juvenile, or any kind of syndromic glaucoma. Glaucoma was diagnosed on the basis of intraocular pressure over 21 mmHg and/or the presence of glaucomatous optic neuropathy and/or glaucomatous changes in perimetry.

Exclusion criteria were other types of glaucoma (closed angle, phacomorphic, augmented episcleral venous pressure, inflammatory, and uveitic glaucoma), a history of ocular hypertension after retinal detachment, intraocular tumour, haemorrhage, postsurgical patients, and trauma.

Hereditary glaucoma/OHT was determined according to the Gong et al. criteria (index case and two affected first degree relatives from consecutive generations) [12]; the remaining cases that did not comply with this definition were classified as familial glaucoma/OHT.

All participating ophthalmologists completed a standardized Excel questionnaire in order to homogenize the collection of epidemiological and clinical data. All patients underwent a full history including systemic and ophthalmologic pathologies, other family members affected by glaucoma (number and type of relative), a family history of consanguinity, and whether they were born in a municipality with <500 inhabitants (to check for masked consanguinity).

Patients were also asked about the places of naissance of their parents and relatives in order to confirm that they were all from Spanish origin.

Patients also underwent a complete ophthalmologic examination including LOG-MAR best-corrected visual acuity, refractive status, slit lamp examination, Goldmann applanation tonometry, and funduscopy. Ancillary tests included computerized perimetry, ultrasound pachymetry, gonioscopy, optic disc photography, and optical coherence tomography (OCT).

Each referring ophthalmologist graded the disease stage, taking into account the ophthalmologic exams, the aspect of the optic disc, and visual field testing results and classified each case as ocular hypertension, initial, moderate, or severe glaucoma. Visual field damage was quantified according to Hodapp-Parrish-Anderson grading scale according to mean defect MD (initial if MD < −6 dB, moderate if MD between −6 and −12 dB, and advanced if MD > −12 dB). Data on the number and type of ocular surgeries (including phacoemulsification) were recorded, as were current ocular hypotensive medicines.

### 2.1. Statistical Analysis

Quantitative variables were described using medians and interquartile ranges [P25, P75] and absolute ranges (minimum and maximum) and qualitative variables using relative frequencies. Groups were compared using the Mann–Whitney *U* test for quantitative variables and Fisher's exact test for qualitative variables. The analysis was made using the SPSS statistical program v20. A type I bilateral error of 5% was applied.

## 3. Results

The results are summarized in Tables 1 and 2. We collected data from 152 index patients included in the EMEIGG study database of unrelated glaucoma families throughout Spain [11]. Of these, 92 (61%) were true hereditary cases and 60 (39%) were familiar cases.

The median age was 64 (46; 75) years, and 83 (54.6%) were female (52 [56.5%] in hereditary patients and 31 [51.7%] in familiar patients). POAG was the most frequent type of glaucoma in both hereditary and familial patients, followed by secondary open angle glaucoma (pseudoexfoliative and pigmentary), congenital glaucoma, and juvenile glaucoma. No significant differences were found between hereditary and familial patients with respect to disease type.

Hereditary cases had a significantly older mean age and more ophthalmologic comorbidity and presented with a lower spherical equivalent but had more surgeries.

There were no significant differences in LogMAR visual acuity values in hereditary and familial patients, although hereditary patients had more advanced perimetry damage (greater negative mean defect values), more severe structural damage to the optic disc (significantly higher proportion of splinter haemorrhages among the other GON factors), and a lower proportion of initial glaucoma cases.

No significant differences were found between familial and hereditary patients in other parameters studied, including intraocular pressure, gonioscopy, anterior segment anomalies, or pachymetry.

Medically, hereditary patients more frequently used first-line drugs, and familial patients second-line drugs. Hereditary patients had a greater frequency of surgical interventions (including phacoemulsification).

The median number of affected relatives was 2 [1; 4] in hereditary patients and 1 [1; 3] in familiar patients (*p* = 0.003). Affected relatives were most frequently parents and offspring, although the only significant relationship was for daughters (*p* = <0.001) (Table 2).

No differences were found between patients born in municipalities with <500 inhabitants or >500 inhabitants.

## 4. Discussion

Familial clustering has been a known risk factor for glaucoma for decades and has gradually gained more weight as a decisive factor for the initiation or not of glaucoma treatment. Initial reports suggested a 30% risk of developing glaucoma in relatives of an index case, but later reports have suggested the risk could be 56% or even 75% [12]. This suggests that POAG has a strong genetic component. The family history is clinically important because the risk of POAG among first-degree relatives of a POAG patient is 7–10 times higher than that of the general population, and surveillance targeting these individuals is indicated for the early detection and treatment of POAG [13].

In the Thessaloniki Eye Study (TES), the prevalence of undiagnosed glaucoma in POAG patients was 57.1%, which was significantly higher than the 34.9% found in pseudoexfoliative glaucoma patients. Other population-based studies throughout the world have reported higher estimated prevalences of undiagnosed glaucoma (60% to 93.3%). The natural history of the disease shows that glaucoma is a silent, asymptomatic disease before advanced damage with significant visual impairment occurs. The TES showed a trend to reduce odds of undiagnosed glaucoma in POAG patients who reported a family history of glaucoma [14].

Weih et al. found that, other than age, the strongest risk factor for glaucoma was a family history of glaucoma and that, in definite glaucoma cases, a family history of glaucoma was the only significant risk factor other than age. However, the authors concluded that reporting of a family history of glaucoma is likely to be underestimated, resulting in an underestimate of the strength of the association between family history and the risk of glaucoma. As in the Baltimore Eye Survey, they found a substantial bias in the reported family history between patients who were diagnosed or undiagnosed at the time of the study. Although not statistically significant, 29% of patients who were already diagnosed reported a family history of glaucoma compared with 15% who were undiagnosed [13, 15]. The study was carried out in urban and rural zones of Australia and found an unexplained higher concentration of glaucoma cases in rural areas [15]. This might possibly be explained by the hypothesis that there were higher rates of masked consanguinity in smaller rural locations. We found no evidence of this in Spain today, but it may have been more plausible in rural Victoria 15 years ago.

In our study, hereditary glaucoma cases were significantly more severe than familial cases and tended to appear in older patients who presented with optic nerves with greater structural and functional damage and required more aggressive medical treatments and more surgeries to control the disease. The median of affected relatives was significantly higher, as expected due to the definitions used.

Potential biases of our study could include the presence of real hereditary cases hidden among familial cases due to the patient's lack of knowledge of affected relatives and the higher rates of ocular surgeries, including phacoemulsification, in hereditary patients. Cataract surgery could have modified other ocular parameters (central visual acuity, pachymetry, etc.) and caused bias (no significant differences in visual acuity between hereditary and familial patients and a lower spherical equivalent in hereditary patients).

A family history of glaucoma is a risk factor that all ophthalmologists should bear in mind when taking decisions on treatment, especially when there is low concordance between the myriad of structural and functional glaucoma tests or progression algorithms currently in use or when there is a borderline result [16]. However, a negative family history of glaucoma does not always imply that it does not exist, as it may be caused by patients' unawareness or confusion with other ocular conditions. The Ocular Hypertension Treatment Study (OHTS) data on family history were collected by patient recall with no verification by chart review or contact with the relatives; thus, this information is likely to be incomplete and incorrect. A family history of glaucoma was not significant in the 2002 OHTS multivariate analysis of risk factors, and this information was not collected in the European Glaucoma Prevention Study (EGPS) Group [17–19].

Screening for glaucoma is difficult, and it has repeatedly been shown that half the patients with glaucoma are undiagnosed and, in at least one study, half of these patients have had an eye examination in the preceding twelve months and had still been missed. One study showed that 97% of patients with undiagnosed glaucoma had a visual field defect, 66% had a vertical-cup-disc ratio of ≥0.7, and 12% had cup asymmetry of ≥0.3 [20]. To date, no single test has been shown to be satisfactory for glaucoma screening, and thus an appropriate strategy would require a combination of several tests. However, the single biggest risk factor for glaucoma is a positive family history. Most people are unaware of whether they have a first-degree relative with a history of glaucoma, and this reflects a breakdown in our responsibility to inform our patients with glaucoma of the importance of the family history. The single biggest impact physicians may have in the detection of undiagnosed glaucoma is to ask all glaucoma patients to inform their first-degree relatives of their substantially increased risk and encourage relatives to inform the specialist at the next eye examination [21].

It is known that glaucoma cases with a positive mutational diagnosis are frequently more severe [22]. In our study, only 5% of POAG cases presented with a detectable mutation, in line with other reports [11, 23]. However, the remaining true hereditary cases are also consistent with the presence of more advanced glaucoma. These cases presented significantly worse structural and functional optic nerve characteristics, more ocular comorbidities, and needed greater amount of medical and surgical treatment to control intraocular pressure with respect to the familiar cases. Therefore, it may be suggested that the more patent the genetic component in a glaucoma case, the worse the prognosis irrespectively of other factors such as age of diagnosis, level of intraocular pressure, and treatment required.

In light of these results, ophthalmologists should first be encouraged to specifically search for a family history of glaucoma when examining a patient and, secondly, to consider more aggressive treatment when there is greater suspicion of a hereditary component.

## Figures and Tables

**Table 1:** Demographic, clinical, and treatment variables in hereditary and familiar glaucoma patients. Spain, 2015.

Group	Hereditary	Familiar	*p* value
Number of cases	92 (61%)	60 (39%)	
Age (Yrs)	66 (46;75)	59 (39;68)	0.007
Sex (female)	52 (56.5%)	31 (51.7%)	0.618
*Glaucoma Subtype*			
POAG	72 (78.3%)	40 (66.7%)	0.335
Secondary	6 (6.5%)	4 (6.7%)	
Juvenile	7 (7.6%)	8 (13.3%)	
Congenital	6 (6.5%)	8 (13.3%)	
Syndromic	1 (1.1%)	0 (0%)	
Severity of glaucoma			
OHT	4 (4.3%)	5 (8.3%)	0.319
Initial glaucoma	19 (20.7%)	22 (36.7%)	0.039
Moderate glaucoma	29 (31.5%)	12 (20%)	0.137
Severe glaucoma	40 (43.5%)	19 (31.7%)	0.174
*Ophthalmic Variables*			
Ophthalmic comorbidities	20 (32.8%)	4 (10%)	0.09
VA (LOGMAR)	0.22 [0.05; 0.4]	0.1 [0; 0.3]	0.067
Spherical equivalent	−0.75 (−2;1)	−1.25 (−3.5; −0.25)	0.046
Optic disk lesions (Splinter Hemorrhages)	15 (16.5%)	3 (5%)	0.04
VF MD	−10.42 [−18; −4.39]	−5.475 [−13.51; −2.78]	0.046
Surgeries	63 (68.5%)	28 (46.7%)	0.01
*Medical treatments*			
Prostaglandin analogs	45 (48.9%)	18 (30%)	0.028
Alpha adrenerics	7 (7.6%)	11 (18.3%)	0.07

YRS: years; OHT: ocular hypertension; VA: visual acuity; VF MD: visual field mean defect; median and range values are provided.

**Table 2:** Reported relatives with glaucoma in hereditary and familiar glaucoma patients. Spain, 2015.

	Hereditary	Familiar	*p* value
Median number of reported relatives with glaucoma (P25; P75)	2 (1; 4)	1 (1; 3)	0.003
Father	29 (31.5%)	7 (11.7%)	0.006
Mother	32 (34.8%)	11 (18.3%)	0.029
Brothers	30 (32.6%)	19 (31.7%)	1
Sisters	29 (31.5%)	15 (25%)	0.465
Sons	24 (26.1%)	8 (13.3%)	0.069
Daughters	19 (20.7%)	0 (0%)	<0.001
Paternal uncles	2 (2.2%)	2 (3.3%)	0.647
Paternal aunts	0 (0%)	2 (3.3%)	0.154
Maternal uncles	3 (3.3%)	0 (0%)	0.278
Maternal aunts	5 (5.4%)	1 (1.7%)	0.404
Paternal grandfather	5 (5.4%)	0 (0%)	0.157
Paternal grandmother	1 (1.1%)	0 (0%)	1
Maternal grandfather	2 (2.2%)	1 (1.7%)	1
Maternal grandmother	2 (2.2%	0 (0%)	0.519
Others	8 (8.7%)	6 (10%)	0.782
